# Novel dataset and model for restroom sound event classification

**DOI:** 10.1038/s41598-025-18154-z

**Published:** 2025-09-01

**Authors:** Ali Emre Öztürk, Erkan Kıymık, Kağan Mehmet Özkök

**Affiliations:** 1 Department of Electrical and Electronics Engineering, Hasan Kalyoncu University, Gaziantep, Turkey; 2 Sanko School, Gaziantep, Turkey

**Keywords:** Sound event detection, Deep learning, Water-usage activities, Semi-supervised learning, RegNetY, Adaptive ensemble model, Electrical and electronic engineering, Computer science

## Abstract

This study presents a novel privacy-preserving deep learning framework for accurately classifying fine-grained hygiene and water-usage events in restroom environments. Leveraging a comprehensive, curated dataset comprising approximately 460 min of stereo audio recordings from five acoustically diverse bathrooms, our method robustly identifies 11 distinct events, including nuanced variations in faucet counts and flow rates, toilet flushing, and handwashing activities. Stereo audio inputs were transformed into triple-channel Mel spectrograms using an adaptive one-dimensional convolutional neural network (1D-CNN), dynamically synthesizing spatial cues to enhance discriminative power. Extensive experimentation identified the RegNetY-008 architecture as the most effective backbone, further improved by employing a semi-supervised learning strategy via pseudo-labeling and targeted data augmentation techniques such as XY masking and horizontal CutMix. The proposed ensemble model, combining RegNetY-008 networks with complementary third-channel generation strategies, achieved outstanding generalization performance, yielding an accuracy of 97.8% and macro-averaged F1-score of 0.966 across acoustically distinct test environments. Our publicly available dataset addresses critical gaps in existing resources, promoting future research in intelligent, privacy-conscious restroom monitoring

## Introduction

Monitoring hand hygiene, water usage, and restroom safety is essential for safeguarding public health, optimizing water resources, and ensuring personal well-being. These concerns are particularly relevant in critical environments such as hospitals, eldercare facilities, and restaurants, where hygiene compliance directly impacts health outcomes, as well as for elderly individuals living alone, who may be especially vulnerable to bathroom-related risks. However, conventional monitoring methods, ranging from manual inspection to camera-based systems and wearable sensors, suffer from cost, scalability, and privacy limitations.

Audio-based monitoring has emerged as a compelling, privacy-preserving alternative. By identifying acoustically distinct restroom events, including toilet flushing, faucet usage (across multiple flow rates and faucet counts), and hand washing, these systems offer scalable solutions for assessing hygiene compliance, water-usage patterns, and potential safety issues. Advances in deep learning and signal processing now enable highly accurate environmental sound classification using spectrogram-based inputs and advanced neural architectures.

This study introduces a robust and generalizable deep-learning framework for audio-based restroom monitoring, capable of classifying 11 fine-grained restroom events involving various faucet configurations, flow rates, hand washing, and toilet flushing. To develop and evaluate our approach, we constructed a comprehensive dataset comprising approximately 460 min of labeled and unlabeled stereo audio recordings from five bathrooms. These environments featured identical faucet and drop-surface configurations but differed significantly in room size and interior layout, thus introducing diverse acoustic characteristics such as reverberation levels, echo patterns, and background noise. Training data were collected exclusively from one bathroom, while testing data originated from another acoustically distinct bathroom. This rigorous partitioning ensures a realistic domain shift for assessing the model’s generalization capabilities. The unlabeled recordings, focused exclusively on faucet operation events, were approximately 1.6 times larger than the faucet-related portion of the labeled dataset and included segments with a randomly varying number of active faucets (1–3), each operating at a single, randomly selected flow rate.

We first converted each stereo recording into Mel spectrograms. We compared them with alternative representations, including MFCCs and power spectra, confirming that Mel spectrograms consistently achieved superior accuracy on our dataset. Since most pre‑trained vision models are designed for three‑channel inputs, we explored different strategies to generate a third spectrogram channel. Beyond simply satisfying the architectural input requirement, we found that the choice of third‑channel strategy also impacts performance. A trainable 1D-CNN that dynamically fused the left and right channels yielded the highest accuracy, while simpler static methods, such as RMS fusion, served as competitive baselines^1^.

To further benchmark our approach, we also evaluated state‑of‑the‑art audio‑specific models directly on raw waveform inputs, including BEATs‑Base, HuBERT‑Base, and wav2vec 2.0‑Base^2–4^. Among these, BEATs‑Base performed best (macro‑F1 = 0.921), but still fell short of our RegNetY‑008 ensemble (macro‑F1 = 0.966) and required far greater computational resources.

To enhance robustness, we applied random segment cropping, XY masking, and horizontal CutMix^5,6^, simulating real‑world acoustic variability. Through ablation studies, we optimized segment length, third-channel strategy, and pseudo-label confidence thresholds, evaluating both spectrogram-based vision models and raw-waveform transformers. RegNetY‑008 emerged as the best trade‑off: it outperformed other lightweight vision backbones (EfficientNet, MobileNet, EfficientViT, ViT)^7–12^ and remained more efficient than BEATs while achieving higher accuracy.

Finally, we constructed an ensemble of two RegNetY‑008 networks—one with the 1‑D‑CNN third channel and one with RMS fusion, the strongest static baseline. Logit‑level averaging improved macro‑F1 from 0.952 to 0.966, with negligible inference overhead.

The primary contributions of this work are:

1. A novel deep-learning framework that accurately classifies 11 distinct restroom events across acoustically varied environments.
2. An adaptive, dynamically trainable third-channel fusion method for stereo audio inputs, further enhanced through ensemble modeling with complementary RegNetY-008 networks.
3. A publicly available dataset consisting of approximately 460 min of labeled and unlabeled stereo restroom recordings from five diverse bathrooms, supporting both supervised and semi-supervised learning.

Existing audio-based restroom monitoring solutions typically lack the granularity required to differentiate between faucet flow rates or multiple faucet configurations, and rarely address significant acoustic variability between training and testing scenarios. Our proposed approach addresses these gaps by integrating advanced deep-learning architectures, adaptive spectrogram fusion techniques, and rigorous domain-shift evaluation strategies supported by our curated dataset.

## Related works

### Sound event detection for restroom and hygiene applications

#### Acoustic monitoring of bathroom activities

Recent research has increasingly focused on the potential of acoustic monitoring to understand and manage activities within bathroom environments. A notable contribution in this area is a two-stage sound event detection method proposed to estimate three common bathroom activities: showering, flushing, and faucet usage^13^. This approach first employs the general sound classification network YAMNet, pre-trained on the extensive AudioSet dataset, to ascertain the presence of a general water sound within the input audio^13^. If a water sound is detected, a modified version of YAMNet, termed W-YAMNet, is triggered to identify the specific activity among showering, flushing, and faucet operation^13^. The rationale behind this two-stage structure is to broadly identify the presence of water-related events before attempting a more granular classification of the specific activity^13^.

Transfer learning with YAMNet is a key aspect of this methodology. It allows the researchers to leverage the pre-trained model’s extensive knowledge of diverse sound events while fine-tuning it for the specific sounds of bathroom activities^13^. Furthermore, the development of W-YAMNet is specifically aimed at accommodating the unique acoustic characteristics of individual bathrooms, recognizing that variations in plumbing, faucet structures, and room materials can significantly influence the soundscape^13^. Experimental evaluations of this method on continuous audio data have yielded promising results, suggesting its potential for real-time monitoring applications^13^. The implications of such a system extend to various domains, including the potential to assess the health and safety of elderly individuals living alone by monitoring their bathroom usage patterns in a non-intrusive manner.

The concept of using sound for bathroom activity monitoring is not entirely new, with earlier studies exploring different approaches^14^. These include systems based on carefully designed Hidden Markov Model (HMM) parameters utilizing Mel-Frequency Cepstral Coefficients (MFCCs) for accurate and robust classification of bathroom sound events^14,15^. More recent investigations have also adopted Convolutional Neural Network (CNN) architectures for this task, reflecting the broader trend of leveraging deep learning in audio analysis^16,17^. These early endeavors recognized the potential of acoustic monitoring to address issues such as understanding personal hygiene behavioral problems and providing support for individuals with dementia^14^. The increasing emphasis on smart-home technologies designed to enhance the safety and well-being of elderly individuals living independently further underscores the relevance of research in this area, particularly given the inherent risks associated with bathroom environments.

A clear trend emerging from these related works is the growing reliance on machine learning, specifically deep learning methodologies, for the automated analysis of sound events occurring in bathrooms. Transfer learning, which involves leveraging the knowledge gained from pre-trained models on large datasets, is also being actively explored to improve the performance and efficiency of these monitoring systems^18–20^. However, a closer examination reveals that many existing studies tend to focus on a relatively limited set of bathroom activities, primarily encompassing flushing, showering, and general faucet usage. This often lacks the granularity to distinguish between different faucet flow rates or the number of faucets operated simultaneously. Furthermore, a consistent underlying motivation across these works is the need to provide privacy-preserving monitoring solutions, making audio a particularly suitable modality for sensitive environments like restrooms where video surveillance is often deemed intrusive and ethically questionable.

#### Audio-based detection of hygiene practices

Beyond the general monitoring of bathroom activities, some research has specifically targeted the audio-based detection of hygiene practices, such as hand washing. While dedicated research papers on this topic within restroom environments appear less prevalent compared to broader bathroom activity monitoring, resources like sound effects libraries offer insights into the acoustic characteristics of such activities. For instance, Soundsnap provides a collection of handwashing sound effects, indicating that this action produces a distinct acoustic signature^21^. This suggests that handwashing generates identifiable sounds, such as the running of water, the rubbing of hands, and the use of towels, which could be detected and classified using audio analysis techniques.

While not directly focused on audio, studies utilizing video-based methods for hand hygiene monitoring also underscore the importance of accurately detecting handwashing actions^22^. These studies highlight the challenges in creating comprehensive datasets for hand hygiene, noting limitations in the number of annotated videos, the diversity of individuals performing the actions, and the variety of backgrounds captured^22^. The need for datasets that include correctly performed and incorrect or illusory handwashing motions is also emphasized^22^. Although these works rely on visual information, they indirectly support the potential of audio as a complementary or alternative modality for hand hygiene detection, particularly in scenarios where video might be less suitable due to occlusion or privacy concerns. The relative scarcity of recent publications specifically dedicated to audio-based detection of handwashing within restroom environments, in contrast to the broader scope of bathroom activity monitoring, suggests a potential area for further research and contribution.

### General water sound classification

#### Acoustic characteristics of water sounds

The classification of water sounds extends beyond the specific context of bathroom activities, encompassing a broader range of acoustic events involving water. As previously mentioned, studies focused on acoustic monitoring of bathroom activities inherently involve the classification of water sounds associated with showering and faucet usage^13^. These works demonstrate the feasibility of distinguishing between water-related actions based on acoustic signatures.

Furthermore, a significant body of research has investigated the acoustic properties of pouring liquids, aiming to infer physical characteristics from the sounds produced^23^. This research explores the relationship between the sound of pouring water and properties such as the liquid level in a container, the shape and size of the container itself, the rate at which the liquid is being poured, and the total time it takes to fill the container^23^. To facilitate this research, a dedicated dataset known as “Sound of Water 50” has been created, featuring a collection of audio-visual recordings of pouring liquids into various containers^23^. This detailed analysis of pouring sounds suggests that water sounds contain a wealth of information and that finer distinctions within bathroom water sounds, such as variations in faucet flow rates, might also be acoustically discernible.

In a related domain, research has explored audio analysis for detecting water leaks in pipes^24^. This work proposes a mechanism that amplifies water flow sounds in pipes and employs deep neural networks to classify between scenarios with and without leaks^24^. This application further illustrates the potential of using acoustic characteristics to identify different states of water flow, reinforcing the idea that variations in flow rates during faucet operation could also be classified based on their unique sound signatures. Generally, the classification of water sounds involves analyzing the time-frequency characteristics of the audio signal, aiming to capture the subtle acoustic variations associated with flow dynamics, turbulence, and the interaction of water with different materials and environments. Deep learning models, trained on appropriately labeled datasets, are increasingly being used to learn these intricate acoustic patterns and to perform accurate classification of diverse water sound events.

#### Datasets for water sound classification

The availability of publicly accessible datasets is crucial for advancing research in water sound classification. As previously mentioned, the “Sound of Water 50” dataset provides a valuable resource specifically for studying the sounds of pouring liquids^23^. However, its focus on pouring might limit its utility for broader water sound classification tasks, particularly those related to the diverse sounds encountered in restroom environments.

The ESC-50 dataset, a widely recognized benchmark for environmental sound classification, includes a broader range of sound categories with some relevance to water sounds, such as “toilet flush” and “pouring water”^25^. While ESC-50 offers a diverse collection of environmental sounds, the number of examples per class (40) might not be sufficient for training highly specialized models capable of distinguishing subtle variations in restroom water events.

AudioSet stands out as a large-scale dataset with a hierarchical ontology encompassing a wide variety of sound events, including several relevant to restrooms, such as “toilet flush,” “water tap, faucet,” “sink (filling or washing),” and “bathtub (filling or washing)”^26^. Its massive scale, comprising thousands of hours of audio, makes it a valuable resource for pre-training deep learning models for various audio tasks, including sound event detection in bathrooms. Notably, AudioSet has been utilized in other research for training models specifically aimed at detecting bathroom sounds^26^. Within AudioSet, the “Toilet flush” class, for example, contains a substantial number of video segments and a significant total duration of audio^27^. However, it’s important to note that AudioSet is weakly labeled, meaning that the labels indicate the presence of a sound event within a 10-second clip but do not provide precise onset and offset times. Additionally, in some cases, AudioSet provides audio embeddings rather than raw audio waveforms, which might affect its direct usability for certain research methodologies.

Beyond structured datasets, sound effects libraries like Uppbeat offer royalty-free shower sounds^28^. SoundBible also provides various restroom-related sound effects under different licenses, including toilet flushing and bathroom sink sounds. These resources indicate the general availability of individual acoustic events related to restrooms, which could be useful for qualitative analysis or augmenting existing datasets. The “Sound of Water” dataset is also publicly available on the Hugging Face platform^29^, providing access to the research focused on pouring sounds. Datasets derived from citizen science initiatives for environmental sound classification also exist and may include “water” as a broad category^30^. However, these datasets often come with challenges related to class prevalence and the signal-to-noise ratio due to recordings being made in uncontrolled real-world environments. In a different domain, the UK Acoustics Network provides a list of open-access underwater acoustics datasets^31^, demonstrating the established practice of curating and sharing datasets for water-related acoustic events in specialized fields. Furthermore, the TAMAGO dataset for acoustic scene classification in home environments includes a “toilet” scene recorded with multiple microphones^32^, offering data that captures the broader acoustic context of a bathroom. Table 1 summarizes a comparative overview of these and other publicly available datasets relevant to water and bathroom sounds, illustrating differences in scope, annotation detail, and data volume.

**Table 1:** Comparison of publicly available datasets for water and bathroom sounds.

Dataset name	Primary focus	Relevant water/bathroom sound classes	Approximate size	availability	Annotation details
Sound of water 50^23^	Pouring liquids	Pouring (hot/cold, different containers)	805 videos	Publicly Available (Hugging Face)	Audio-visual, labeled for physical properties (liquid level, container dimensions, etc.)
ESC-50^25^	Environmental sound classification	Toilet flush, pouring water	2000 clips (5 s each)	Publicly Available (GitHub)	50 classes, 40 examples per class, manually labeled
AudioSet^26^	Large-scale audio event tagging	Toilet flush, water tap/faucet, sink (filling/washing), bathtub (filling/washing), liquid, drip, steam, etc	> 5000 h	Publicly Available (Google Research)	Weakly labeled (presence/absence in 10 s clips), hierarchical ontology, sometimes provides embeddings instead of raw audio
TAMAGO^32^	Acoustic scene classification (home)	Toilet	~ 2000 h	Not specified	21 acoustic scenes, recorded with 42 microphones

The review of related works reveals a growing interest in leveraging audio-based monitoring for restroom environments, driven by the need for privacy-preserving and scalable solutions for hygiene compliance, water conservation, and safety. The field has experienced a significant shift towards deep learning techniques for sound event detection and classification tasks within restrooms, as well as for broader water sound analysis. Existing studies have demonstrated the feasibility of detecting general bathroom activities such as flushing, showering, and faucet usage, and even inferring physical properties from various water sounds.

However, a notable limitation remains the lack of comprehensive, publicly available datasets specifically tailored for the fine-grained classification of multiple hygiene and water usage events in restroom environments. While datasets like AudioSet and ESC-50 include relevant classes, they typically lack the granularity needed to distinguish among different faucet flow rates or configurations, which is critical for detailed restroom monitoring. Moreover, recent approaches employing transfer learning with networks such as YAMNet suffer from methodological limitations regarding dataset partitioning. Specifically, prior work has often performed training and evaluation within the same acoustic environment, consequently failing to properly assess model generalization to unseen bathroom conditions.

Our study directly addresses these gaps by introducing a robust deep-learning framework capable of accurately classifying 11 distinct hygiene and water-usage events evaluated across acoustically diverse restroom environments. Furthermore, the creation and utilization of our meticulously curated dataset, consisting of approximately 460 min of labeled and unlabeled stereo restroom recordings from five acoustically varied bathrooms, represent significant contributions to the field. By employing a strict, environment-based data partitioning strategy—where training and testing data originate from entirely separate bathroom environments—we ensure a rigorous evaluation of our model’s real-world generalization capabilities. Combining methodological rigor with practical applicability, our work advances the state-of-the-art in environmental sound classification, offering a privacy-conscious solution for intelligent restroom monitoring.

## Materials and methods

### Dataset collection

#### Bathroom selection and acoustic variability

Audio data were collected from five different bathrooms at Hasan Kalyoncu University, each selected to reflect diverse spatial and acoustic environments. While all bathrooms utilized the same faucet (battery) models and identical falling surfaces, they varied significantly in overall area, the number of installed faucets, and the geometric arrangement of those faucets. Differences in room dimensions and surface materials also contributed to unique reverberation patterns and sound reflection behaviors. This diversity in acoustic settings allows for a comprehensive evaluation of our audio classification models under realistic and varied environmental conditions.

#### Recording setup and microphone placement

**Fig. 1:**
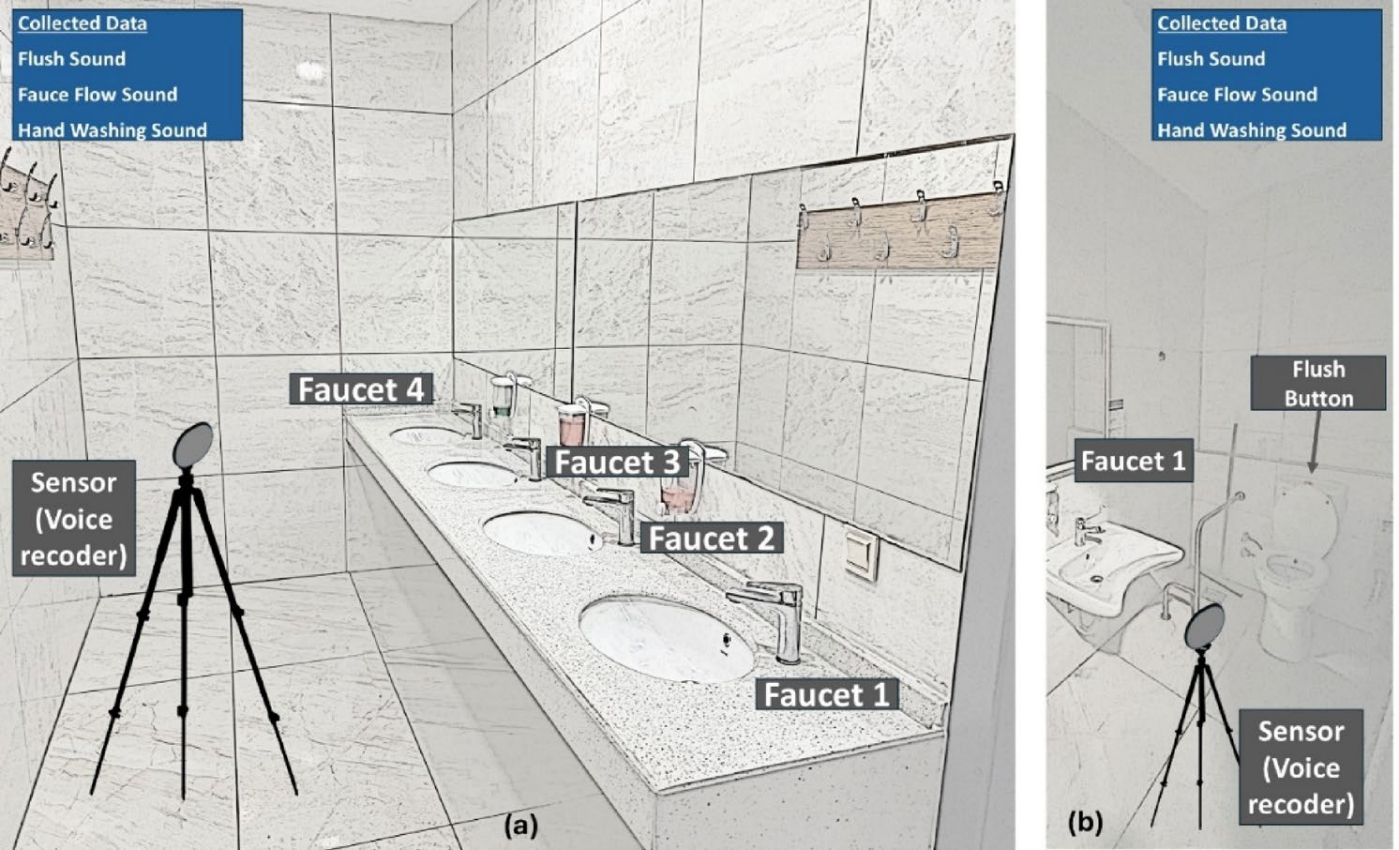
(**a**) One of the audio recording environment visuals for multiple—single faucet, flush, hand washing sounds. (**b**) One of the audio recording environment visuals for single faucet, flush, hand washing sounds.

Recordings were made using five identical audio recorders, each configured to capture stereo audio signals. All recordings were saved in WAV format with a sampling rate of 16 kHz and a bitrate of 512 kbps. Recorders were positioned based on the number and arrangement of faucets in each bathroom to ensure consistent and realistic data collection. In bathrooms with a single faucet, the microphone was symmetrically placed in front of the faucet at varying distances between 30 and 40 cm. In setups with two faucets spaced 60 cm apart, the microphone was centrally positioned between them. The microphone was placed directly in front of the middle faucet when three faucets were present. This systematic placement strategy maintains consistency while still capturing natural acoustic variability introduced by differences in room geometry and faucet configuration. Figure 1 illustrates the recording setups, showing microphone placements for single and multiple faucet configurations, as well as flush and handwashing events.

#### Class definitions and data annotation

We defined eleven specific audio event classes based on faucet operation, flow rate intensities, and hygiene-related events:

1 Faucet with Flow Rate #1, 1 Faucet with Flow Rate #2, 1 Faucet with Flow Rate #3, 2 Faucets with Flow Rate #1, 2 Faucets with Flow Rate #2, 2 Faucets with Flow Rate #3, 3 Faucets with Flow Rate #1, 3 Faucets with Flow Rate #2, 3 Faucets with Flow Rate #3, Hand Washing, Flush Sound.

Table 2 below presents the flow rates corresponding to the three faucet intensity levels (flow rates 1, 2, and 3) used in this study. For clarity, each rate is expressed in multiple standard units, including cubic centimeters per second (cm^3^/s), liters per minute (L/min), and gallons per minute (gpm).

**Table 2:** Flow rate definitions in multiple Units (cm^3^/s, L/min, gpm).

Label	cm^3^ s^−1^	mL s^−1^	L min^−1^	gal min^−1^ (gpm)
Flow rate 1	8.31472	8.31	0.50	0.13
Flow rate 2	32.00288	32.00	1.92	0.51
Flow rate 3	71.592	71.59	4.30	1.14

Each faucet and flow rate combination was recorded for at least 7 min for both training and testing, totaling a minimum of 14 min per combination. Audio events were manually labeled by annotators who marked each event’s start and end timestamps within the recorded audio files. Additionally, we collected supplementary unlabeled audio data to support semi-supervised learning methods.

#### Dataset distribution and partitioning

To rigorously assess the generalization capability of our audio classification model, we employed a strict environment-based partitioning strategy. Specifically, labeled data for model training were collected exclusively from Bathroom A, while labeled test data originated from the acoustically distinct Bathroom B, ensuring clear domain separation. This design avoids the same-environment leakage reported in prior YAMNet studies and yields a more realistic generalization benchmark. The labeled dataset, which is approximately 740 MB, includes around 500 MB of faucet operation audio segments with explicitly defined flow rates and counts. Additionally, we acquired an unlabeled dataset of roughly 850 MB consisting exclusively of faucet operation recordings from all five bathrooms (A–E), with randomly varying numbers of simultaneously active faucets (ranging from 1 to 3) and randomly selected flow rates per segment. This extensive unlabeled dataset provides substantial acoustic variability, encompassing diverse reverberation characteristics, room geometries, and background noises, facilitating effective semi-supervised learning and enhancing the model’s robustness to unseen acoustic environments.

### Preprocessing and feature extraction

We employed a preprocessing pipeline to convert raw audio recordings into discriminative feature representations suitable for deep learning-based environmental sound classification.

#### Stereo audio transformation and feature representation

**Fig. 2:**
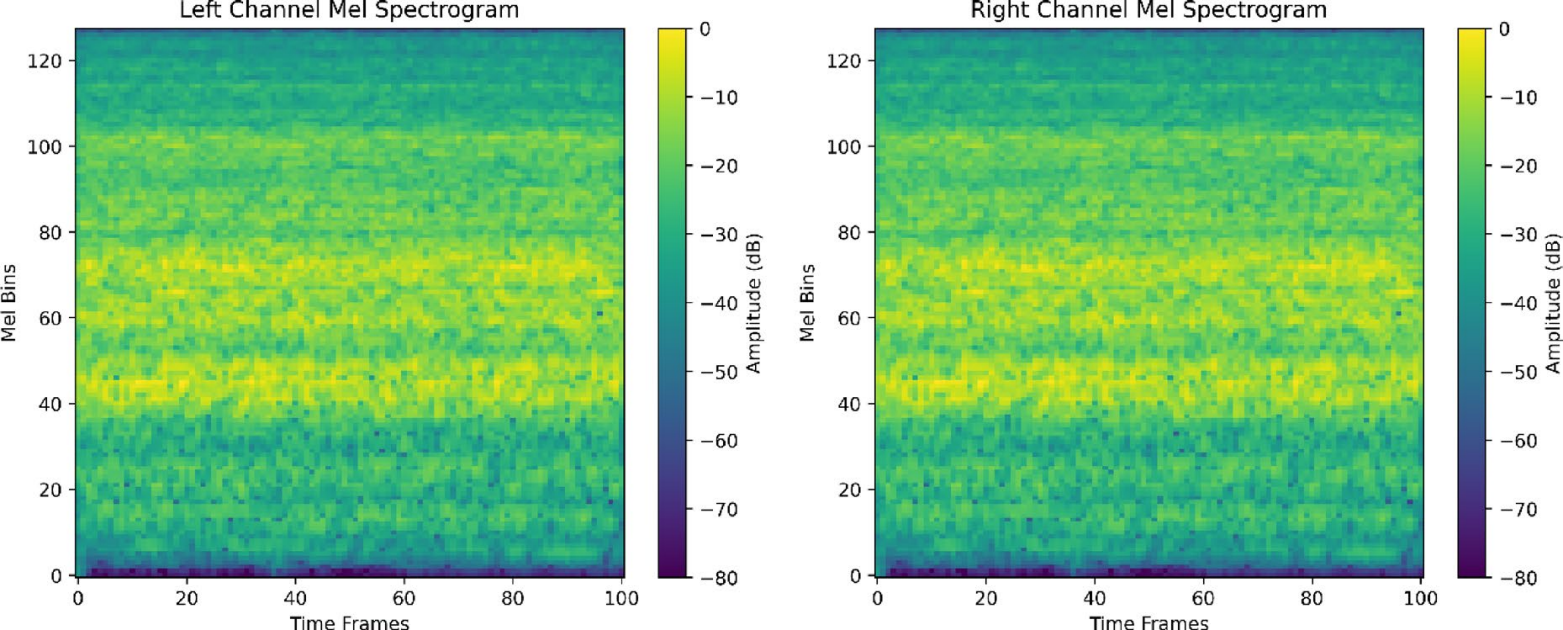
Left and Right Channel Mel Spectrograms Extracted from a Stereo Audio Segment.

All recordings were captured in stereo format at a sampling rate of 16 kHz. To preserve temporal context, each audio file was segmented using a sliding‑window approach. We compared three spectral representations, Mel spectrograms, MFCCs, and power spectra, under identical experimental settings and observed that Mel spectrograms consistently outperformed the alternatives in terms of classification performance (Table 4). Accordingly, Mel spectrograms were adopted as the primary feature representation, given their demonstrated effectiveness in modeling both spectral and temporal characteristics of acoustic events ^33^. Figure 2 illustrates example left and right channel Mel spectrograms extracted from a stereo audio segment.

Mel spectrograms were generated using empirically optimized parameters: a segment duration of 3.0 s with a 1.5-s overlap, a sampling rate of 16 kHz, an FFT window size of 512, a hop length of 160, and 128 Mel frequency bins. The frequency range was limited to 20–8000 Hz, and the power spectrogram was computed using a power exponent of 2.0.

Segment durations of 1, 3, and 5 s were systematically evaluated, with a 3-s window and 1.5-s overlap yielding the best trade-off between temporal resolution and contextual coverage. The hyperparameter search space comprised:

- n_fft: {256, 512, 1024}, hop_length: {128, 160, 256}, n_mels: {64, 128, 256}
- fmin: {0, 20, 50}, fmax: {4000, 8000, 12000}, power: {1.0, 2.0}

##### Dynamic third-channel generation from stereo inputs

We transformed the stereo Mel spectrograms into a three-channel format to align with the input requirements of standard pre-trained vision-based architectures, such as RegNetY-008. Several third-channel generation strategies were investigated.

Static aggregation techniques, including minimum, maximum, mean, and RMS were initially explored due to their simplicity and low computational cost. However, their fixed nature limited the model’s ability to adapt to complex acoustic variations.

We implemented a learnable third-channel generation mechanism based on a 1D-CNN to address this limitation. During training, this module dynamically synthesized the third channel by learning optimized feature transformations from the left and right channels. The adaptive nature of this strategy enabled the model to capture nuanced audio cues, such as subtle distinctions in water flow intensity and hygiene-related sounds, across diverse spatial configurations.

Overall, integrating optimized Mel spectrograms with a dynamically trainable third-channel formulation provided a robust and generalizable input representation, enhancing the downstream classification performance in acoustically heterogeneous restroom environments. Beyond satisfying the three-channel input requirement, the learnable 1D-CNN exploits inter-aural level/time differences; empirically, it lifts the macro-F1.

### Model architecture

To determine the most suitable architecture for our audio classification task, we extensively evaluated multiple deep-learning models spanning various design paradigms. These included traditional CNNs, Temporal convolutional networks (TCNs)^34^, and a range of state-of-the-art pre-trained models^35^.

#### Evaluated model architectures

**Fig. 3:**
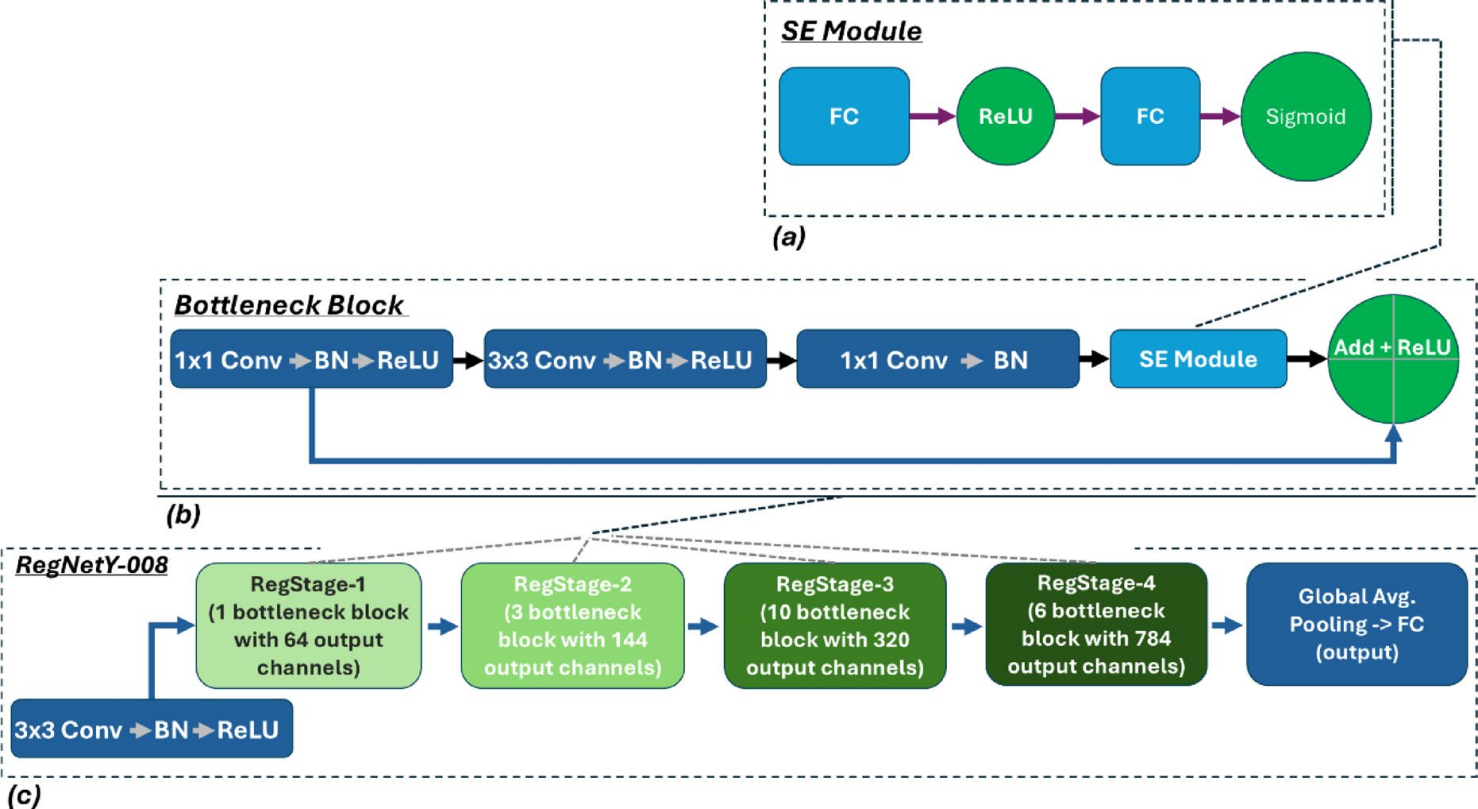
(**a**) Structure of the Squeeze-and-Excitation (SE) module. (**b**) Internal Structure of a Bottleneck Block with Integrated SE Module. (**c**) RegNetY-008 architecture overview: stem, four RegStages, global pooling, and classifier.

The pre-trained architectures evaluated in this study included:

- *EfficientNet* Utilizes compound scaling of depth, width, and resolution to achieve improved accuracy with reduced parameter count and computational demand^8,36^.
- *MobileNet* Employs depthwise separable convolutions, offering lightweight and fast inference performance suitable for edge devices^9^.
- *TinyNet* Designed for minimal resource usage, offering strong performance in constrained environments^37^.
- *MnasNet* Derived from neural architecture search, optimized for latency and efficiency^38^.
- *MixNet* Incorporates mixed depthwise convolutions, balancing representational capacity and efficiency^39^.
- *EfficientViT_B0* Combines CNN and ViT components to extract local and global features efficiently^40^.
- *HuBERT (Base)* A self-supervised transformer model that learns audio representations by predicting clustered pseudo-labels of masked waveform segments. It combines a convolutional encoder with a transformer to capture both local and global acoustic patterns, achieving strong performance on speech and audio tasks^3^.
- *wav2vec 2.0 (Base)* A self-supervised transformer model that learns audio representations by predicting masked latent features from raw waveforms. It combines a CNN encoder for feature extraction with a transformer for context modeling, achieving strong performance on speech and audio tasks^4^.
- *BEATs* is a self-supervised transformer model for audio representation learning. It uses a two-stage pretraining: first, an acoustic tokenizer converts audio into discrete tokens; then, a transformer learns to predict masked tokens. Combining a CNN frontend with transformer layers, BEATs achieves strong performance on audio and speech tasks^2^.
- *RegNetY-002* Lightweight CNN from the RegNetY family with stage-wise bottleneck blocks and Squeeze-and-Excitation (SE) modules. Optimized for efficiency with low computational cost.
- *RegNetY-016* Mid-sized RegNetY model using residual bottleneck blocks with SE modules. Balances accuracy and complexity for scalable performance.
- *RegNetY-008* RegNetY-008 is a convolutional neural network architecture derived from the RegNet family, built on simple stage-wise design principles^7^. The network is organized into a stem (initial convolution), a body with four stages of residual blocks, and a final classification head. Each stage consists of a fixed number of bottleneck blocks with identical output widths, and successive stages use increasing channel widths in a roughly linear progression. The internal structure of each bottleneck block is illustrated in Fig. 3b, highlighting the sequence of convolutions, normalization, activation functions, the integrated Squeeze-and-Excitation module, and the residual connection.

For the selected RegNetY-0.8GF variant (RegNetY-008), the network consists of four stages producing output channel widths of 64, 128, 320, and 768, respectively. These stages contain [1–3, 8] bottleneck blocks, resulting in a total depth of 14 learnable layers. Each block employs a fixed bottleneck ratio of 1:1 (no internal channel reduction) and a consistent group width of 16 channels per group, thus forming a structured, ResNeXt-style design with grouped convolutions. Additionally, RegNetY integrates a Squeeze-and-Excitation (SE) attention module into each bottleneck block (RegNetY = RegNetX + SE), facilitating effective channel-wise feature recalibration^41^. Figure 3a illustrates the detailed architecture of the SE module, highlighting its global pooling operation followed by two fully connected layers that perform adaptive channel gating.

We chose the RegNetY‑0.8 GF (RegNetY‑008) variant because it offers the best trade‑off between capacity, efficiency, and inductive bias for our 460‑minute restroom‑acoustics dataset: (i) Moderate depth and width (≈ 5.4 M parameters, 0.8 GFLOPs) are large enough to model fine‑grained spectral patterns yet small enough to avoid overfitting on a modest‑sized corpus. (ii) RegNetY’s grouped depthwise convolutions and SE blocks act as channel‑attention mechanisms, enabling the network to emphasise frequency bands that are diagnostic of specific water‑usage events. (iii) The architecture’s linear stage‑width scaling yields smoothly expanding receptive fields that match the gradual temporal evolution of faucet‑flow spectra while still capturing abrupt events such as toilet flushes. In a pilot comparison (Table 5), RegNetY‑008 outperformed the lighter RegNetY‑002 (− 0.07 F1) and the heavier RegNetY‑016 (− 0.02 F1). These considerations, balanced accuracy, computational cost, and an attention mechanism aligned with the task’s frequency‑selective nature, motivated its selection as the backbone for our proposed system.Finally, the overall structure of the RegNetY-008 architecture is presented in Fig. 3c. These design choices, a shared bottleneck ratio and group width across layers and linearly increasing stage widths, originate from a principled search of the RegNet design space^7,12^. They result in a simple yet flexible architecture that can be tuned for different model sizes while maintaining high efficiency and representational power.

All candidate architectures were evaluated under consistent conditions, with preprocessing adapted to each model type. Vision‑based models, including RegNetY‑008, EfficientNet, and others, were trained on Mel spectrograms, MFCCs, and power spectrograms, while audio‑based transformers like BEATs and wav2vec 2.0 were fine‑tuned on raw audio. Performance was measured by accuracy and macro‑averaged F1.

#### Triple-channel input configuration

As most pre-trained models require three-channel input (mirroring RGB image data), we adopted a stereo-to-triple-channel strategy. The left and right channels of the stereo Mel spectrogram served as the first two input channels. The third channel was generated through two alternative methods:

1. *Static Aggregation (RMS)* Computed as the root mean square of the left and right channels.
2. *Trainable 1D-CNN* A dedicated 1D convolutional network was trained to learn a dynamic third channel representation from the stereo inputs, updated via backpropagation during model training.

The use of a trainable third channel enhanced adaptability and allowed the model to extract richer representations of spatial and temporal audio dynamics. Table 3 summarizes the optimal configuration of the 1D-CNN architecture used for third-channel generation, detailing the layer types, dimensions, kernel sizes, and activation functions employed to synthesize discriminative feature representations from stereo inputs.

**Table 3:** Architecture details of the trainable 1D-CNN for third-channel generation.

Layer no	Layer type	Input channels	Output channels	Kernel size	Padding	Activation
1	Conv1d	2	8	7	3	ReLU
2	Conv1d	8	16	5	2	ReLU
3	Conv1d	16	1	3	1	ReLU

#### Classification head and ensemble strategy

The classification head of each model comprised a global average pooling layer followed by fully connected layers aligned with the number of target classes. All models were trained using cross-entropy loss^42^ and softmax activation^43^. To further improve classification performance, we employed an ensemble approach combining two RegNetY-008 models:

- *Model A* RegNetY-008 with the third channel generated via trainable 1D-CNN.
- *Model B* RegNetY-008 with the third channel derived using RMS aggregation.

**Fig. 4:**
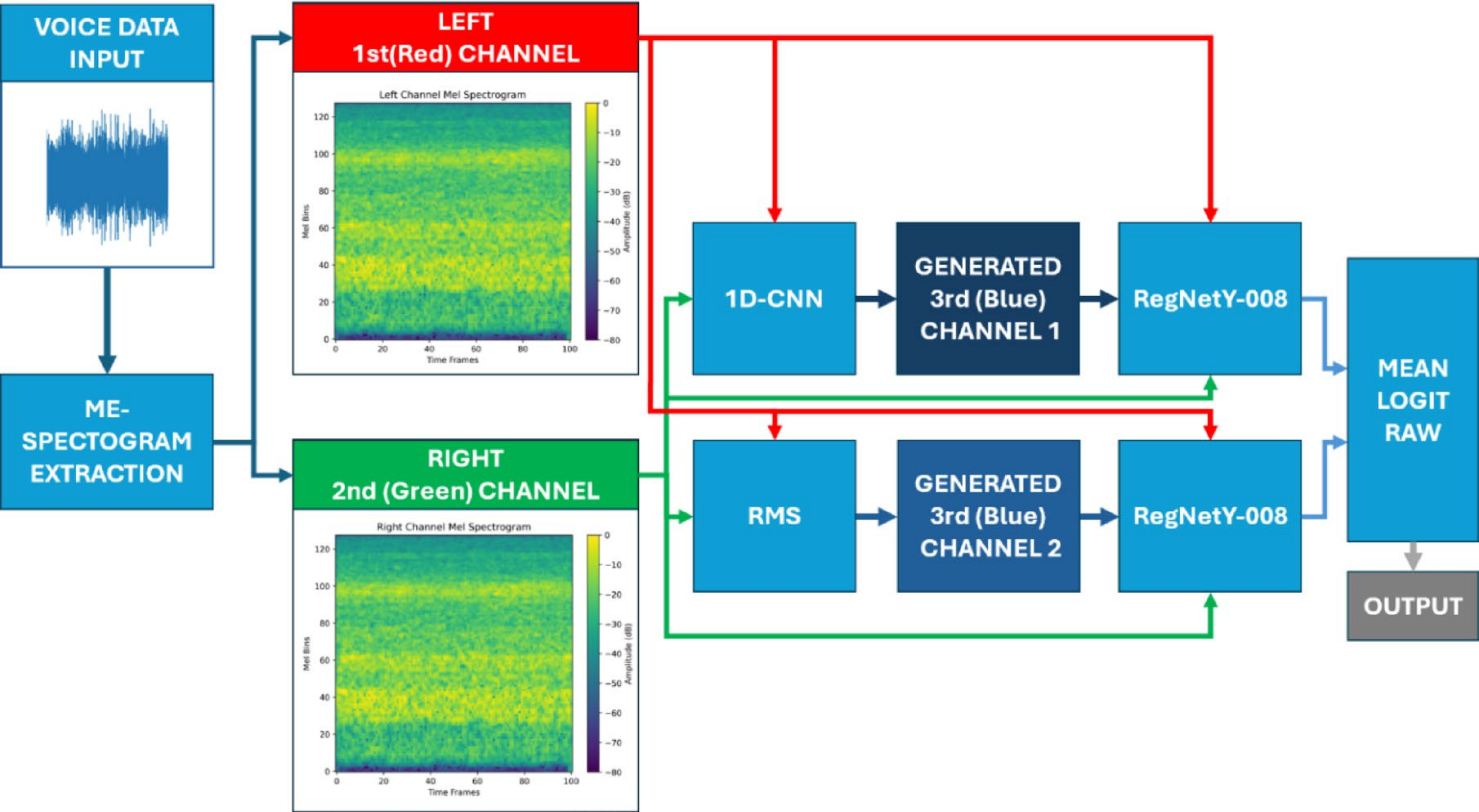
Ensemble model combining RegNetY-008 with 1D-CNN and RMS-based third-channel inputs.

Figure 4 shows the overall structure of the ensemble model, which integrates two RegNetY-008 networks using different third-channel generation methods, 1D-CNN and RMS fusion.

During inference, we averaged both models’ raw logits (pre-softmax outputs) to produce final predictions. This mean-logits ensemble strategy effectively leveraged the complementary strengths of the static and dynamic third-channel approaches, resulting in enhanced accuracy and stability across diverse test conditions.

This architecture, featuring RegNetY-008, robust triple-channel input generation, and ensemble inference, constituted the foundation of our final model and demonstrated consistently superior performance throughout our experimental evaluation.

### Training strategy

This section outlines the training procedures employed in our study, which include advanced data augmentation techniques, a semi-supervised pseudo-labeling strategy^44,45^, and an optimization framework tailored for robust and accurate audio classification.

#### Data augmentation

To improve model generalizability and mitigate overfitting, we implemented several data augmentation strategies specifically designed for spectrogram-based audio classification tasks:

##### Random audio segment transform

**Fig. 5:**
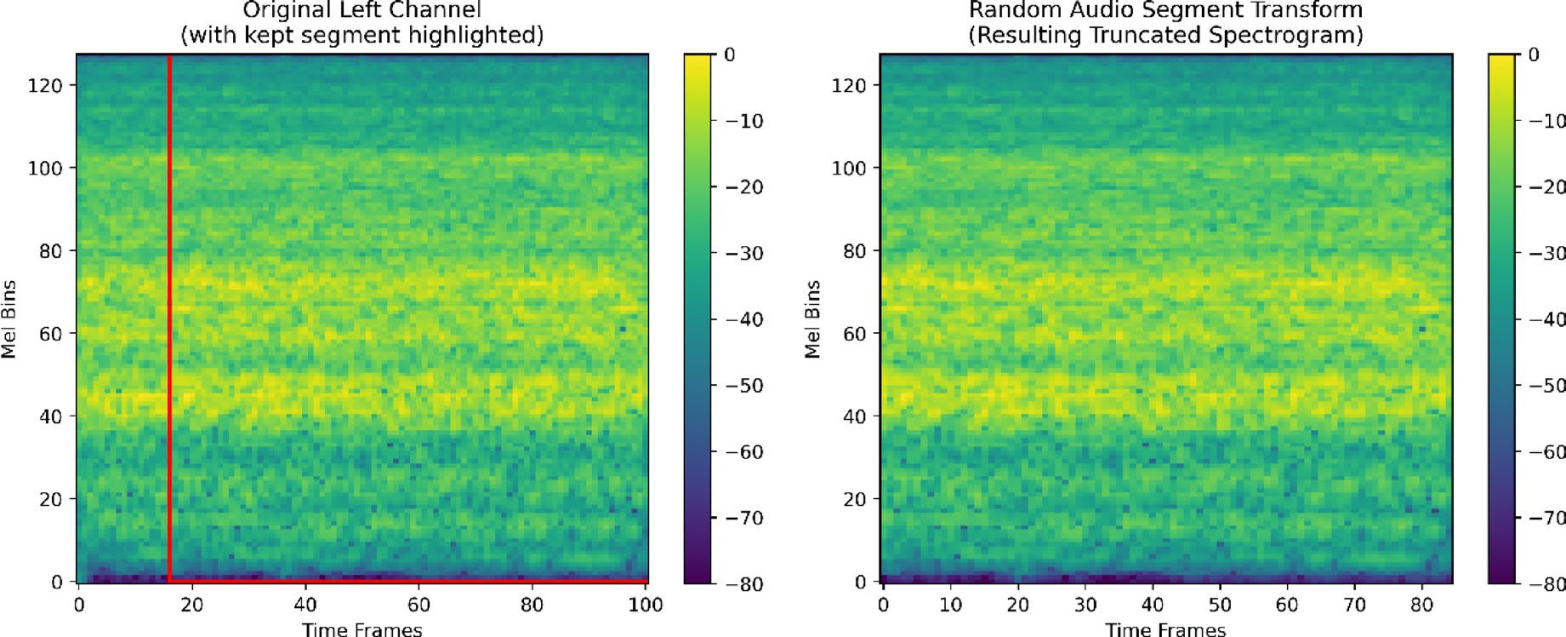
Example of random audio segment transform applied to a Mel spectrogram.

We applied a probabilistic segmentation. With a probability of 0.5, each input tensor was randomly truncated to retain between 75 and 90% of its original length. This augmentation encourages robustness to temporal variability and simulates partially captured or truncated events. An example visualization of this augmentation is shown in Fig. 5.

##### XY masking transform

**Fig. 6:**
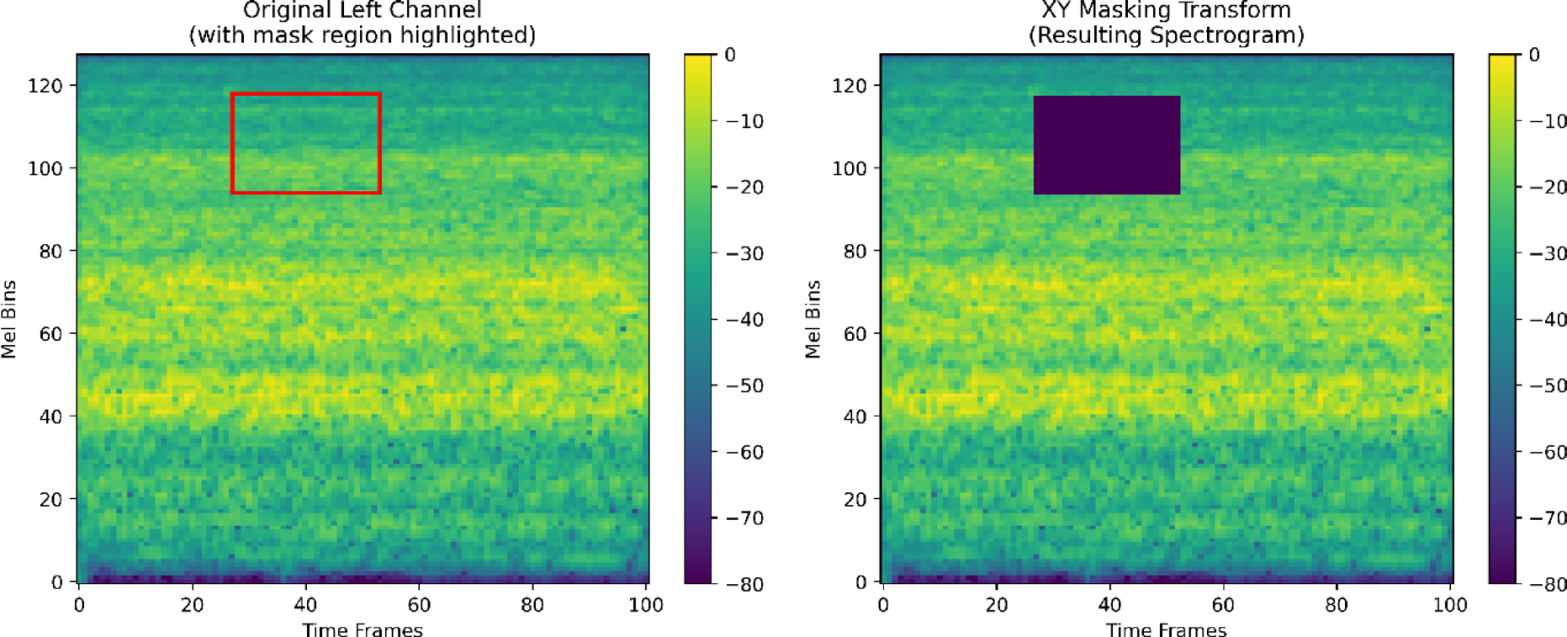
Example of XY masking transform applied to a Mel spectrogram.

Time–frequency masking was applied, where rectangular patches covering 10% to 30% of the spectrogram were randomly masked with a probability of 0.5. This approach, similar in principle to SpecAugment^46^, forces the model to learn invariant features and enhances its resilience to localized noise or missing data. Figure 6 illustrates an example of the XY masking effect.

##### Horizontal CutMix

**Fig. 7:**
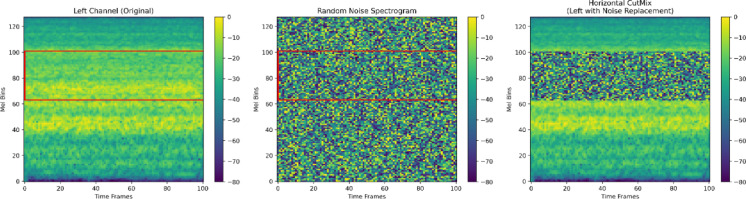
Visualization of Horizontal CutMix applied to a Mel spectrogram.

Inspired by the CutMix strategy originally developed for image classification, we implemented a horizontal variant that replaces a contiguous horizontal band in the spectrogram with the corresponding band from another randomly selected sample in the same batch. The mixing ratio is dynamically determined by sampling a cut ratio from a uniform distribution between 0.2 and 0.5 of the spectrogram height. This augmentation promotes learning from incomplete or occluded patterns and improves the model’s ability to generalize in cases of acoustic overlap. For visualization purposes, the right channel of the spectrogram was replaced with random noise to clearly demonstrate the effect of Horizontal CutMix, as illustrated in Fig. 7.

Collectively, these augmentation methods enrich the training distribution, introduce variability in the temporal and spectral domains, and prepare the model for real-world deployment conditions with unpredictable noise and occlusion.

#### Semi-supervised learning and pseudo-labeling strategy

**Fig. 8:**
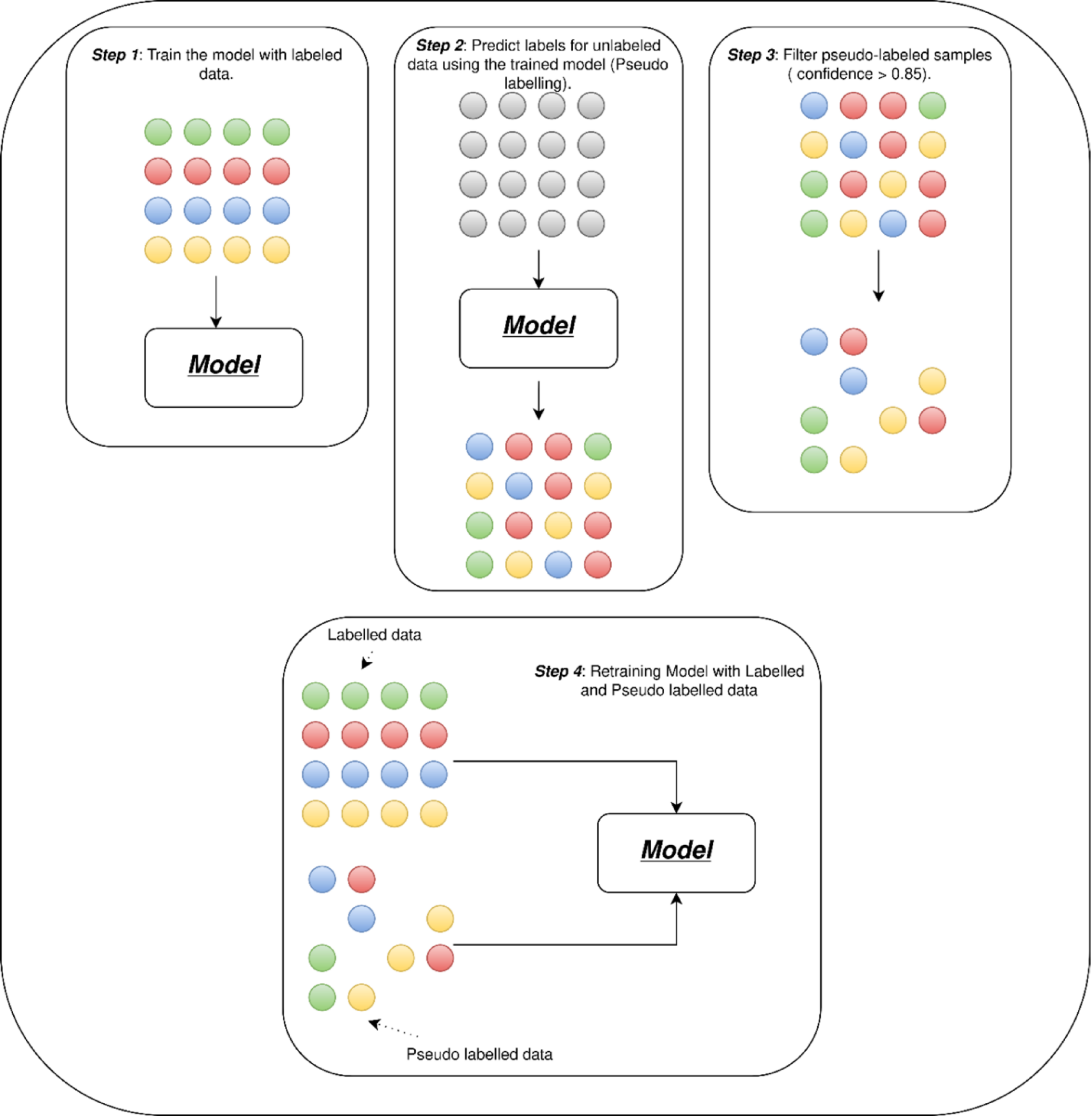
Diagram of the semi-supervised learning strategy based on pseudo-labeling.

To further exploit the unlabeled portion of the dataset, we employed a semi-supervised learning strategy based on pseudo-labeling. After initial supervised training on the labeled dataset, the trained model was used to predict labels for unlabeled samples. Figure 8 presents a diagram illustrating the Semi-Supervised Learning and Pseudo-Labeling Strategy.

A confidence threshold was applied to determine the acceptance of these predictions as pseudo-labels. We empirically tested thresholds in the range [0.75, 0.95], ultimately selecting 0.85 as the optimal value based on validation accuracy and test set performance. Only predictions with class confidence ≥ 0.85 were retained and used as additional training data. This iterative pseudo-labeling process augmented the training set and enhanced the model’s ability to generalize without requiring manual annotation of the unlabeled audio.

#### Loss function and optimization

The model was trained using the cross-entropy loss function, which is well-suited for multi-class classification problems^42^.

Optimization was carried out using the AdamW optimizer, which decouples weight decay from gradient updates and offers improved regularization over standard Adam^47^. Additionally, the cosine annealing learning rate scheduler was implemented to modulate the learning rate over epochs^48^. This scheduler gradually reduces the learning rate following a cosine decay pattern, promoting stable convergence and helping the model escape local minima.

**Fig. 9:**
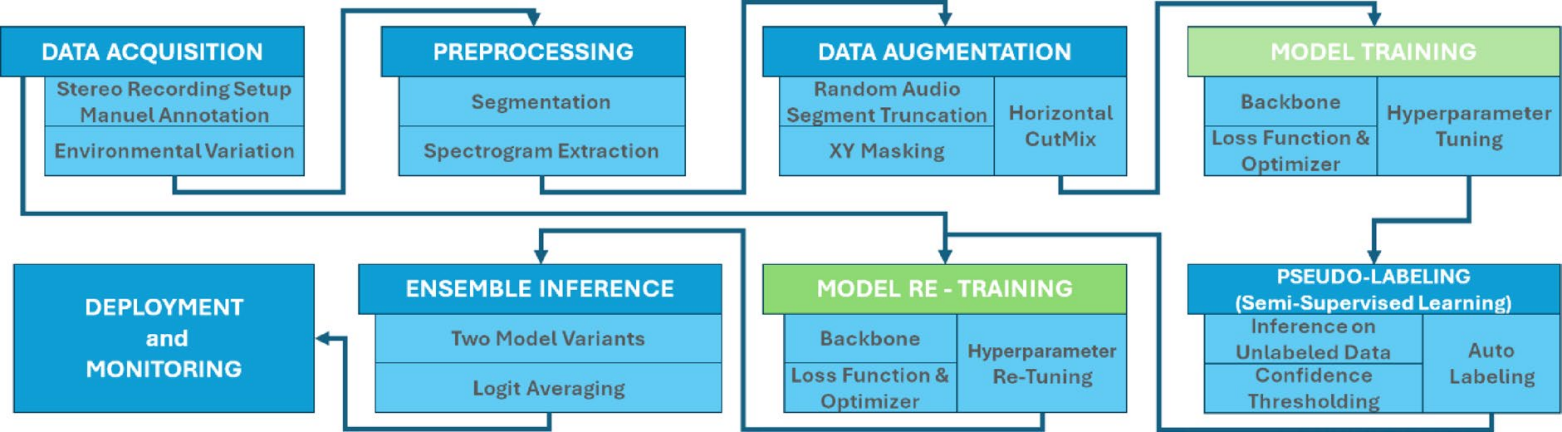
Complete system pipeline from data acquisition to deployment.

Figure 9 illustrates the complete system pipeline, from data acquisition through model training, pseudo-labeling, and deployment.

## Results

This section presents and analyzes the performance of our audio classification framework under various design choices, including backbone architecture, segment lengths, channel-generation methods, and pseudo-label confidence thresholds. Each subsection details experiments and findings that guided the selection of the final optimized model.

### Visual-based models (spectrogram input)

We first evaluate models trained on time–frequency representations of audio, treating the task as a visual recognition problem. We compare different input features (Mel spectrograms, MFCCs, and power spectrograms), backbone architecture, segment durations, stereo channel fusion strategies, and pseudo-labeling thresholds to optimize the spectrogram-based approach.

#### Feature comparison

To achieve optimal performance with the RegNetY-008 model, the features listed in Table 4 were systematically evaluated. Among these, the Mel spectrogram representation yielded the highest accuracy and F1-macro scores, demonstrating its effectiveness for the acoustic classification task.

**Table 4:** RegNetY-008 Performance comparison with different features.

Features- (3rd channel mean)	Accuracy	F1-macro
Mel spectrogram	**0.8825**	**0.8784**
Mel-frequency cepstral coefficients (MFCC)	0.8225	0.8353
Power spectrogram	0.6522	0.6318

Significant values are in (bold).

#### Model architecture comparison (Mel spectogram)

We benchmarked eleven models using 3-s Mel spectrogram inputs without semi-supervised enhancements to identify the most successful model for classifying restroom-related audio events. The models included nine pre-trained architectures (RegNetY variants, EfficientNet, MobileNet, TinyNet, MnasNet, EfficientViT, and MixNet), along with custom CNN and TCN models trained from scratch with optimized hyperparameters. Table 5 presents the accuracy and macro F1 scores for each model on the test set.

**Table 5:** Performance comparison of pretrained backbone models using 3-second Mel spectrograms.

Model- (3rd channel mean)	Accuracy	F1-macro
RegNetY-008	**0.8825**	**0.8784**
MobileNetV2_100	0.8684	0.8500
EfficientNet-Lite0	0.8666	0.8468
RegNetY-002	0.8510	0.8460
MnasNet_A1	0.8243	0.8081
MixNet_S	0.8311	0.7806
CNN(Optimized)	0.8134	0.7723
EfficientViT_B0	0.8429	0.7706
TinyNet_A	0.8114	0.7680
RegNetY-016	0.7880	0.7640
TCN(Optimized)	0.7823	0.7554

Significant values are in (bold).

RegNetY-008 emerged as the clear leader from these results, offering the highest overall accuracy and F1-macro. MobileNet and MnasNet provided competitively lower parameter counts but consistently lagged in performance. Despite their proven success in standard image tasks, MixNet, TinyNet, and EfficientNet also fell short of RegNetY-008’s accuracy.

#### Ablation on clip-length segmentation

Next, we investigated how the temporal segment length of Mel spectrograms influences classification performance, which is crucial for balancing context capture and computational overhead. We compared 1-s, 3-s, and 5-s segments, each processed under the same RegNetY-008 backbone and initial hyperparameter settings. The results are collated in Table 6.

**Table 6:** Effect of segment duration on classification performance using RegNetY-008.

Segment duration (3rd channel mean)	Accuracy	F1-macro	Train Data	Test Data
1 s	0.7833	0.7535	12,453	11,996
3 s	**0.8899**	**0.8548**	4127	4001
5 s	0.7366	0.6794	2478	2387

Significant values are in (bold).

Overall, 3-s segments offered the most effective balance between capturing sufficient temporal context, particularly for events like toilet flushing, and minimizing the inclusion of irrelevant background noise or redundant frames. While 5-s segments occasionally benefited from longer-duration events such as extended flush sound activity, they also increased the likelihood of overlapping frames and introduced confusion in recognizing shorter events. In contrast, 1-s segments often failed to encapsulate complete acoustic signatures, resulting in truncated representations that hindered the accurate detection of longer water-usage events.

#### Triple-channel input approaches

The collected audio is stereo (two channels), whereas the pre-trained networks we employ accept three-channel inputs. We therefore evaluated two simple third-channel generation strategies.

Mono duplication: duplicating a single channel across all three inputs yielded an accuracy of 0.818 and an F1-macro of 0.800.

Zero-padded stereo: keeping the original left and right channels and padding the third channel with zeros improved accuracy to 0.884 and F1-macro to 0.858.

These results confirm that retaining stereo cues enhances performance, motivating our exploration of more sophisticated methods to improve the metrics further.

With the segment length fixed at 3 s, we evaluated various strategies for generating the third input channel from stereo audio. Table 7 shows test accuracy and F1 scores for the following channel-generation approaches:

- Min, max, mean, RMS, trainable 1D-CNN of left and right channels

Static transformations like min, max, mean, and RMS did yield moderate gains, suggesting stereo channel fusion is beneficial. However, the trainable 1D-CNN consistently outperformed these fixed operations, indicating the network’s ability to learn an optimal stereo combination per event. This dynamic approach contributed significantly to improved detection of classes prone to subtle left–right variations, such as multi-faucet configurations.

**Table 7:** Performance comparison of third-channel generation strategies using RegNetY-008 with Fixed 3-second segments.

3rd channel	Accuracy	F1-macro
1D-CNN	**0.9256**	**0.9189**
RMS	0.9060	0.8683
Mean	0.8798	0.8605
Max	0.8494	0.8532
Min	0.8729	0.8528

Significant values are in (bold).

#### Pseudo-label threshold variation

**Fig. 10:**
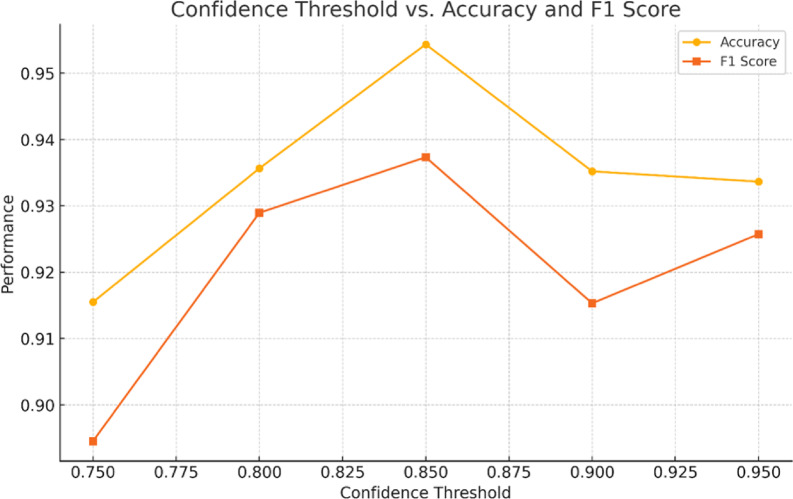
Effect of confidence threshold on classification accuracy and F1 score during pseudo-labeling.

Finally, we explored semi-supervised learning via pseudo-labeling. As described in “Semi-Supervised Learning and Pseudo-Labeling Strategy” Section, unlabeled samples were labeled automatically if the model’s confidence surpassed a chosen threshold. To determine a suitable value, we trialed thresholds in the range [0.75, 0.95] in increments of 0.05. Table 8 presents the resultant accuracy and macro F1 for these settings, while Fig. 10 provides a visual comparison to highlight performance trends across different confidence thresholds.

**Table 8:** Effect of pseudo-label confidence threshold on classification accuracy and F1 score using RegNetY-008.

Confidence threshold (3rd channel 1D-CNN)	Accuracy	F1-macro
0.75	0.9154	0.8945
0.80	0.9236	0.9113
**0.85**	**0.9543**	**0.9403**
0.90	0.9352	0.9153
0.95	0.9266	0.9257

Significant values are in (bold).

Our findings revealed a clear peak near 0.85, striking the best balance between including informative unlabeled samples and excluding unreliable predictions. Lower thresholds introduced noise in the pseudo-labels, resulting in misclassification reinforcement, while higher thresholds were too conservative, yielding fewer useful augmented samples.

### Audio-based models (raw waveform input)

In addition to visual-based methods, we investigated models capable of learning directly from raw audio waveforms without explicit feature extraction. These audio-based models leverage transformer architectures pre-trained on large audio corpora, enabling them to capture rich temporal and spectral patterns. This section compares the performance of state-of-the-art audio models and examines the effect of semi-supervised pseudo-labeling when applied to raw waveform inputs.

#### Model architecture comparison (raw-audio)

We evaluated three state‑of‑the‑art transformer‑based models on 3‑second raw waveform inputs: BEATs‑Base, HuBERT‑Base, and wav2vec 2.0‑Base. All models were fine-tuned on the restroom audio dataset to evaluate their effectiveness for end-to-end audio classification.

As shown in Table 9, BEATs-Base achieved the best results, with an accuracy of 92.04% and an F1-macro of 92.12%, substantially outperforming both HuBERT-Base and wav2vec 2.0-Base. These findings underscore the effectiveness of BEATs-Base for raw waveform classification in this application domain.

**Table 9:** Performance comparison of raw-audio models fine-tuned on restroom audio events.

Model (3 s raw waveform)	Accuracy	F1-macro
BEATs	0.9204	0.9212
HuBERT base	0.7346	0.6944
Wav2vec 2.0 base	0.6771	0.6843

#### Pseudo-label threshold variation

We applied semi-supervised learning to the best-performing raw-audio model, BEATs, by incorporating pseudo-labeled samples into the training process. As described in “Semi-Supervised Learning and Pseudo-Labeling Strategy” Section, only unlabeled samples with predicted confidence above a specified threshold were added.

Table 10 summarizes the results for confidence thresholds ranging from 0.75 to 0.95. The BEATs model achieved the highest performance at a threshold of 0.85, yielding an accuracy of 93.63% and an F1-macro of 93.16%.

**Table 10:** Effect of pseudo-label confidence threshold on BEATs model performance.

Confidence threshold (BEATs -3 s raw waveform)	Accuracy	F1-macro
0.75	0.8954	0.8985
0.80	0.9136	0.9113
**0.85**	**0.9363**	0.9316
0.90	0.9212	**0.9345**
0.95	0.9210	0.8999

Significant values are in (bold).

### Backbone selection: BEATs versus RegNetY-008 + 1D-CNN

We compared the two best-performing backbones: BEATs, trained on raw waveform inputs, and RegNetY-008 with 1D-CNN third-channel fusion, trained on 3-s Mel spectrograms. As shown in Table 11, BEATs achieved the highest F1-macro (0.9212), slightly outperforming RegNetY-008 + 1D-CNN (0.9189).

**Table 11:** Performance comparison of pretrained backbone models using 3-second Mel spectrograms.

Model	Params (M)	Speed advantage	F1-macro
BEATs	90	–	0.9212
RegNetY-008 (1D-CNN)	5.4	2.85 × faster	0.9189

However, BEATs comes with significantly higher computational cost, with 90 million parameters, compared to RegNetY-008’s lightweight 5.4 million parameters and 0.8 GFLOPs. Furthermore, RegNetY-008 offers approximately 2.85 × faster inference, making it more suitable for deployment on resource-constrained and edge devices where latency and efficiency are critical.

Considering this trade-off, we selected RegNetY-008 with 1D-CNN third-channel fusion as the backbone for our ensemble model, as it balances competitive performance with superior computational efficiency.

### Final model performance

The final model performance was evaluated through an ensemble approach, combining two independently trained RegNetY-008 models. These models differed solely in the method used for third-channel generation: one utilized a trainable 1D-CNN, while the other employed a fixed RMS aggregation. Model predictions were combined using mean logits to form the ensemble output.

Table 12 summarizes the comparative performance metrics, including accuracy and macro F1-score, for the ensemble model and its individual component models. The ensemble demonstrated superior performance, indicating effective complementarity between the two approaches.

**Table 12:** Final performance comparison of individual and ensemble RegNetY-008 models.

Model	Accuracy	F1-macro
RegNetY-008 3rd channel RMS	0.9278	0.9132
RegNetY-008 3rd channel 1D-CNN	0.9532	0.9439
Ensemble model	**0.9783**	**0.9662**

Significant values are in (bold).

**Fig. 11:**
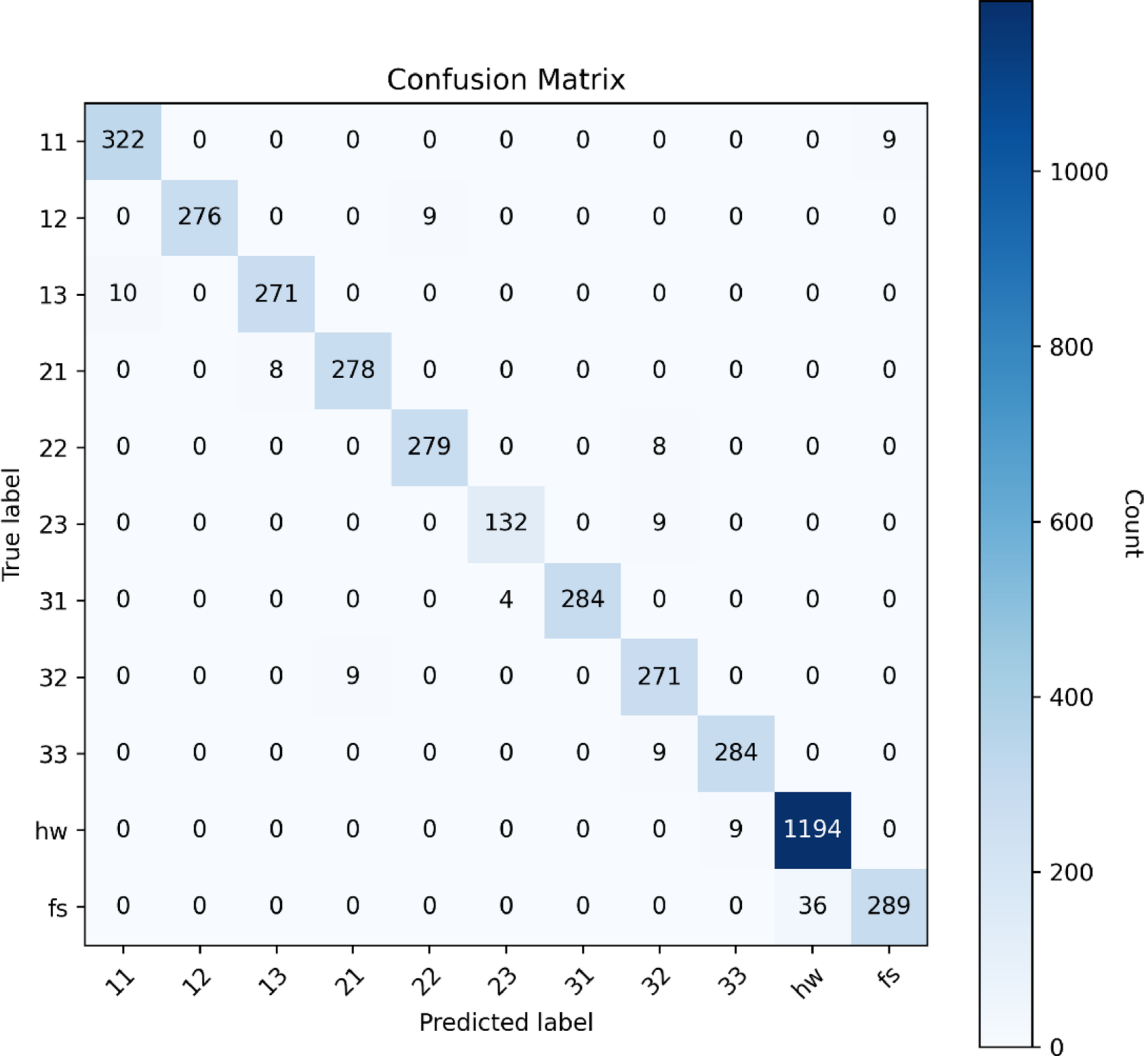
Confusion matrix for the ensemble model on the test set.

A confusion matrix analysis for the ensemble model (Fig. 11) highlights robust recognition for dominant event categories, such as flush, hand washing, and single-faucet flows. Classification errors occurred primarily among closely related multi-faucet flow categories (e.g., flow rates 2 and 3), reflecting acoustic similarities between these classes.

As presented in Table 9, the final ensemble model achieved an overall test accuracy of 97.8% and a macro-averaged F1 score of 0.966, indicating strong and consistent performance across all 11 sound event classes. Most faucet-related categories (e.g., classes 11–33) achieved F1 scores exceeding 0.96, highlighting the model’s capability to accurately differentiate subtle variations in faucet count and flow rate. The highest F1 score was observed for handwashing (hw) at 0.981, while flush sounds (fs) exhibited slightly lower performance (F1 = 0.928), likely due to greater acoustic variability. The class labels follow a structured naming convention, where the first digit denotes the number of active faucets (1–3) and the second digit indicates the flow rate level (1–3), as detailed in Table 13.

**Table 13:** Per-class precision, recall, and F1 scores for final ensemble model on test set.

Class	Precision	Recall	F1-score	Support
11	0.9699	0.973	0.9714	332
12	0.9684	0.9684	0.9684	285
13	0.9713	0.9644	0.9678	279
21	0.9693	0.9713	0.9703	287
22	0.9688	0.9721	0.9710	288
23	0.9706	0.9362	0.9530	136
31	0.9693	0.9861	0.9770	293
32	0.9713	0.9679	0.9696	280
33	0.9693	0.9693	0.9693	293
hw	0.9707	0.9925	0.9810	1230
fs	0.9701	0.8892	0.9280	298
Accuracy	0.98			4001
Macro avg	0.96991	0.96276	0.96607	4001
Weighted avg	0.97006	0.97072	0.97005	4001

## Discussion

This study presents a robust, privacy-preserving, audio-based monitoring framework capable of classifying 11 distinct restroom events, including nuanced differences in faucet count and flow intensity. The proposed approach advances state-of-the-art environmental sound classification through architectural innovation, dataset design, and domain-aware training strategies.

### Model performance and generalization

Our ensemble RegNetY-008 model achieved an accuracy of 97.8% and a macro-averaged F1-score of 0.966 across acoustically distinct test environments. These strong performance metrics demonstrate the model’s generalization ability under real-world domain shifts. The training and test sets were collected from different bathrooms with varying reverberation patterns, geometries, and background noise, ensuring a rigorous evaluation protocol. Importantly, the model successfully distinguished acoustically similar classes, such as multiple faucet configurations operating at adjacent flow levels, validating the discriminative power of our spectrogram encoding and adaptive third-channel design.

The choice of RegNetY-008 as the backbone was motivated by its favorable trade-off between capacity, efficiency, and inductive bias for fine-grained acoustic classification. Its moderate depth and width (5.4 M parameters, 0.8 GFLOPs) were sufficient to model subtle spectral patterns without overfitting, while its grouped depthwise convolutions and SE modules acted as effective channel-attention mechanisms, enhancing frequency-selective discrimination. In pilot experiments, RegNetY-008 outperformed both lighter (RegNetY-002, − 0.07 F1) and heavier (RegNetY-016, − 0.02 F1) variants, confirming its suitability for this task.Compared to prior studies that focused on broader restroom categories such as flushing, hand washing, or undifferentiated faucet usage, our work introduces finer-grained classification by capturing spatial and intensity-related nuances. This is made possible by transforming stereo Mel spectrograms into triple-channel inputs using a learnable 1D-CNN, which outperformed static fusion methods. The adaptive nature of this learned representation allowed the model to better capture spatial audio cues essential for class separation.

### Impact of semi-supervised learning and augmentations

Another key strength of our framework lies in its ability to leverage unlabeled data through a confidence-based pseudo-labeling strategy. Using a threshold of 0.85, we selectively incorporated high-confidence predictions from unlabeled data gathered across five bathrooms, introducing additional acoustic variability into training. This semi-supervised pipeline improved model robustness and reduced reliance on manual annotation.

Data augmentation also played a critical role. Techniques such as random segment cropping, XY masking, and our novel horizontal CutMix variant improved the model’s resilience to occlusions, ambient noise, and variable sound patterns. Additionally, as shown in Table 4, Mel-spectrograms outperformed alternative acoustic features, such as MFCC and power spectrum, achieving higher macro-F1 and accuracy. These methodological choices enabled the model to generalize well to ambiguous or overlapping acoustic events, further validating the real‑world applicability of our design.

### Comparison to existing works

When compared to existing literature, particularly models based on YAMNet or conventional CNNs, our approach exhibits higher precision and significantly broader event coverage. Prior studies typically classify a limited set of restroom events without differentiating between faucet configurations or flow rates. Moreover, many rely on weakly labeled or synthetic data, which limits their generalizability. Most of these studies train and evaluate within the same bathroom, so their accuracies reflect in‑bathroom repeatability rather than cross‑environment robustness. Our model, by contrast, is trained on one restroom and tested on an acoustically distinct restroom, achieving 0.97 macro‑F1 despite this domain shift.

In contrast, we introduce a curated and publicly shareable dataset of labeled and unlabeled stereo recordings from five acoustically diverse restrooms. This dataset fills a crucial gap by enabling fine-grained hygiene activity classification under real-world noise conditions and variable room layouts. By releasing this dataset to the research community, we aim to accelerate future progress in privacy-aware audio monitoring for hygiene and water management applications.

### Audio-specific transformer models

We fine-tuned three state-of-the-art waveform transformers to provide audio-focused baselines. BEATs reached a macro-F1 of 0.921, outperforming HuBERT-Base (0.694) and wav2vec 2.0-Base (0.684). However, BEATs requires ~ 90 M parameters and triples inference time relative to our RegNetY-008 ensemble, which attains a higher macro-F1 of 0.966 with just 5.4 M parameters. Thus, when accuracy and efficiency are jointly considered, the vision-based RegNetY approach remains preferable, although BEATs is a compelling option when compute budget is unconstrained.

### Real-world implications

The proposed system has strong implications in healthcare, eldercare, and smart-home settings. It can passively monitor hand hygiene compliance, detect abnormal usage patterns (e.g., prolonged faucet use), and support safety interventions, all while preserving user privacy. Importantly, the system functions without video surveillance, making it suitable for sensitive environments where visual monitoring is ethically or practically infeasible.

### Limitations and future work

Despite the promising, several limitations remain. The current dataset, while diverse, covers only five bathrooms from a single institution. To better assess generalization, future work should include a broader range of environments, such as public restrooms, commercial kitchens, and buildings with varied architectural and plumbing styles.

Classification errors persist among classes with overlapping acoustic signatures, particularly for multi‑faucet events at similar flow levels. Exploring complementary sensing modalities, such as low‑resolution thermal imaging or contactless vibration sensing, may help resolve such ambiguities while preserving privacy.

Finally, real‑time deployment aspects, including latency, energy consumption, and edge feasibility, were beyond the scope of this study. Future work could also explore optimizing audio-based transformer models, such as BEATs, to reduce their computational overhead, or hybrid approaches that combine their temporal resolution with the efficiency of lightweight vision-based models, like RegNetY‑008. These directions are essential for enabling scalable, on-device integration in IoT-enabled smart environments.

## Conclusion

This study presents a robust and privacy-conscious deep learning framework for audio-based restroom monitoring. It is capable of classifying 11 distinct hygiene and water-usage events, including nuanced faucet configurations and flow rates, with high accuracy and generalization. Leveraging stereo audio recordings transformed into Mel spectrograms, the proposed system introduces a novel third-channel generation strategy, where a trainable 1D-CNN adaptively fuses spatial audio cues to improve discriminative capacity.

Our final ensemble model, combining two RegNetY-008 networks with complementary third-channel inputs, achieved an accuracy of 97.8% and a macro-averaged F1-score of 0.966 under strict domain shift conditions where training and testing environments were acoustically distinct. The integration of semi-supervised learning through pseudo-labeling, together with targeted data augmentation techniques, further enhanced model robustness without requiring additional human annotation.

Importantly, we contribute a curated, labeled, and unlabeled stereo restroom audio dataset collected across five bathrooms with diverse acoustic characteristics. This dataset will be made publicly available, providing the research community with a valuable resource for advancing privacy-preserving sound event detection in real-world settings.

The practical implications of this work are significant. The system can support hygiene compliance monitoring, water usage analysis, and safety detection in environments such as hospitals, eldercare facilities, and smart homes, without relying on cameras or wearable devices, thereby ensuring user privacy.

We plan to expand the dataset for future work across varied institutions, architectural designs, and usage scenarios. Exploring multi-modal extensions with non-visual sensors may also help disambiguate acoustically similar classes, pushing the boundaries of privacy-aware behavior monitoring.

## Data Availability

The audio data collected by our team within the scope of this study will be shared as an open source via the link below. The data was collected specifically for this study and is the first data created. https://github.com/aliEmreOzturk/water_leakage_voice_data.

## Abbreviations

1D-CNN: One-dimensional convolutional neural network
BEATs: Bidirectional encoder representation from audio transformer
CNN: Convolutional neural network
ESC-50: Environmental sound classification dataset (50 classes)
FFT: Fast Fourier transform
HMM: Hidden Markov model
HuBERT: Hidden-unit bidirectional representation transformer
IoT: Internet of Things
MFCCs: Mel-frequency cepstral coefficients
RMS: Root mean square
SE: Squeeze-and-excitation
TCN: Temporal convolutional network
ViT: Vision transformer
YAMNet: YouTube AudioSet-Based Model for Sound Classification
